# Systemic Lupus Erythematosus and Pulmonary Hypertension

**DOI:** 10.3390/ijms24065085

**Published:** 2023-03-07

**Authors:** Konstantinos Parperis, Nikolaos Velidakis, Elina Khattab, Evangelia Gkougkoudi, Nikolaos P. E. Kadoglou

**Affiliations:** Medical School, University of Cyprus, Nicosia CY2024, Cyprus

**Keywords:** systemic lupus erythematosus (SLE), pulmonary arterial hypertension (PAH), pulmonary hypertension (PH), biomarkers, medical treatment, prognosis

## Abstract

Pulmonary Hypertension (PH) is a common manifestation in patients with Systemic Lupus Erythematosus (SLE) and varies from asymptomatic to life-threatening disease. PH can result not only from immune system dysregulation, but also from various conditions, including cardiorespiratory disorders and thromboembolic diseases. Most commonly, SLE-related PH presents with non-specific symptoms, such as progressive dyspnea on exertion, generalized fatigue and weakness and eventually dyspnea at rest. Prompt diagnosis of SLE-related PH and early identification of the underlying pathogenetic mechanisms is demanded in order to introduce targeted therapy to prevent irreversible pulmonary vascular damage. In most cases the management of PH in SLE patients is similar to idiopathic pulmonary arterial hypertension (PAH). Furthermore, specific diagnostic tools like biomarkers or screening protocols, to establish early diagnosis seem to be not available yet. Although, the survival rates for patients with SLE-related PH vary between studies, it is evident that PH presence negatively affects the survival of SLE patients.

## 1. Introduction

Systemic lupus erythematosus (SLE) is a chronic autoimmune disease of unknown etiology that can affect any body’s organ. Its global prevalence ranges from 13 to 7713.5 per 100,000 individuals, depending on the geographical region while the prevalence in the USA is estimated to be 72.8 per 100,000 individuals according to data derived from a recent meta-analysis and registries [1,2]. SLE is significantly more frequent in women than in men; however, men tend to be diagnosed with the disease at a more advanced age [3]. The diagnosis can be established using the patient’s clinical manifestations and laboratory findings after alternative diagnoses have been excluded. Various classification criteria have been proposed which can aid the diagnosis and include the 1997 American College of Rheumatology criteria, the 2012 Systemic Lupus International Collaborating Clinics criteria and the 2019 European Alliance of Associations for Rheumatology criteria [4].

The clinical manifestations of the disease consist of constitutional symptoms such as fatigue, fever, weight loss or weight gain. Furthermore, the disease can affect virtually any organ of the body and may present with a wide variety of clinical features, including arthritis or arthralgias, mucocutaneous lesions with the characteristic butterfly rash, alopecia, oral ulcers, cardiac involvement, vasculitis, Raynaud’s phenomenon, nephritis, gastrointestinal manifestations, neuropsychiatric symptoms, and hematologic abnormalities.

Pulmonary involvement is also a common manifestation (50–70% of SLE patients) and varies from asymptomatic to life-threatening disease. It may present with interstitial lung disease (ILD), acute pneumonitis, pleural effusion, diffuse alveolar hemorrhage, pulmonary hypertension (PH), pulmonary embolism, pulmonary vasculitis and rarely with shrinking lung syndrome [5,6]. PH is defined as an elevated pulmonary artery pressure (resting mean pulmonary artery pressure (mPAP) above 20 mmHg), measured during right heart catheterization [7]. In SLE patients, PH can be associated with SLE per se, classified to group1 of pulmonary arterial hypertension (PAH). Besides this, it may result from various conditions, including left ventricular dysfunction, congestive heart failure, ILD, or chronic thromboembolic disease, particularly in patients with coexisting antiphospholipid syndrome. Most commonly, PH presents with non-specific symptoms such as progressive dyspnea on exertion, generalized fatigue and weakness and eventually dyspnea at rest [8]. Gradually, signs and symptoms of right heart failure develop, namely elevated jugular pressure, hepatomegaly, ascites and lower extremity edema making apparent the full clinical syndrome [9]. 

A literature search was performed on MEDLINE and EMBASE, Cochrane, and Google Scholar databases for English-language publications. We also checked the reference lists of the identified articles to find any additional relevant articles. Our search included the titles, abstracts, and medical subject headings (MeSH), and we used the following search terms or abbreviations: pulmonary hypertension (PH), pulmonary arterial hypertension (PAH), systemic lupus erythematosus (SLE), epidemiology, pathophysiology, biomarkers, management, therapy, prognosis. Two investigators (N.V. and E.K.) performed the literature search independently. In our search, we included clinical studies (both clinical investigations and clinical meta-analyses). Experimental studies were used only for pathophysiology explanation. To draw firm conclusions, we excluded the studies fulfilling the following criteria: unavailable full texts, publication language other than English, conference abstracts. Using the abovementioned terms, we initially found 1158 hits. After the abstracts’ screening, we removed 23 duplicated studies, and also 984 irrelevant studies; 151 full-text studies were screened for eligibility. After removing the studies with wrong design, irrelevant outcomes, unavailable full text, we ended up with a total of 66 studies, 3 systematic reviews and meta-analyses of clinical data, and 26 additional clinical studies which were not included in those meta-analyses.

The aim of this article is to review the pathophysiology of PH in patients with SLE, the diagnostic strategy focusing on the variety of biomarkers and to summary the therapeutic management, which may alter the final prognosis.

## 2. Epidemiology and Classification of PAH in SLE Patients

During the first World Symposium on Pulmonary Hypertension, PH was defined as an mPAP above 25 mmHg measured by right heart catheterization at rest. Currently, the hemodynamic definition for pre-capillary PH, also known PAH, has set the cut-off value to be >20 mmHg for mPAP combined with a pulmonary vascular resistance (PVR) > 2 Wood units and, pulmonary arterial wedge pressure (PAWP) ≤ 15 mmHg [7]. This change in the diagnostic criteria may result in earlier detection of individuals with pulmonary hypertension. It has been proposed that certain populations with PH may benefit from earlier initiation of therapy [7].

PH is a rare syndrome, classified into five groups: (1) PAH, (2) PH due to left heart disease, (3) PH due to lung disease and/or hypoxia, (4) PH due to pulmonary artery obstructions and lastly (5) PH with unclear or multifactorial mechanisms. PAH can be idiopathic or associated with a wide variety of causes such as connective tissue disorders (CTD) including SLE hereditary, drug or toxins, infectious pathogens [7]. The prevalence of PH in SLE patients ranges between 0.5% and 17.5% [10,11]. It is attributed to either idiopathic causes (50%), or advanced ILD with lung fibrosis, thromboembolic disease, pulmonary veno-occlusive disease and heart disease (50%) [9]. Hence, half of PH cases among SLE patients are attributed to immunological disorders per se while the rest are secondary to cardio-respiratory and vascular diseases. Prospective observational studies suggest female gender and child-bearing age as risk factors for PH development in SLE patients [10,12]. A recent observational study identified the following clinical conditions and laboratory findings highly associated with PH incidence in SLE patients: Raynaud’s phenomenon, digital vasculitis, pericardial effusion, pulmonary interstitial lesions, presence of anti-U1 ribonucleoprotein and anticardiolipin IgG antibody [13]. Interestingly, positive anti-U1 ribonucleoprotein had an independent correlation with PH severity, while pericardial effusion was associated with a longer duration of PH and worse prognosis. Additional risk factors of PH development have been proposed by several other studies [14,15,16,17,18].

## 3. Pathophysiology

PH is an uncommon, but severe complication of SLE and its exact etiopathogenesis remains unknown. Genetic predisposition, environmental stimuli and immune system dysregulation play a key role in the etiology of SLE-related PH [19]. There are also several possible underlying pathogenic mechanisms, including cardiovascular, respiratory and thromboembolic disorders. The hemodynamic and clinical consequences become obvious when pulmonary vascular resistance (PVR) elevates and right heart failure appears leading eventually to death [13,20].

**Figure 1:**
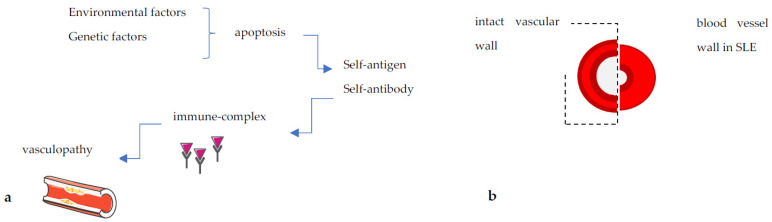
(**a**) Schematic pathophysiology of SLE leading to vasculopathy, including environmental and/or genetic dysregulations. (**b**) Vessel wall in patient with a SLE compared to the intact vascular wall.

**Figure 2:**
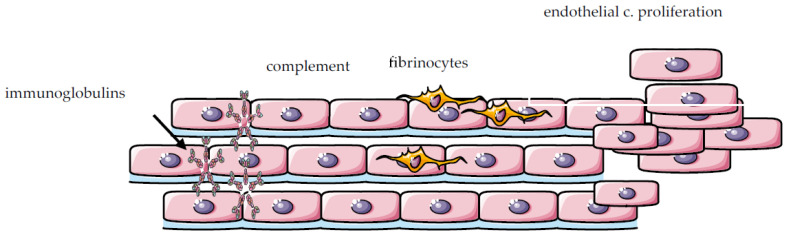
Schematic pulmonary vessel wall and magnification of histopathological features included endothelial and smooth muscle cell proliferation, fibrinoid vasculitis and deposition of immunoglobulins and complement components in the intimal and medial layers of the pulmonary vessels in patients with SLE-related PAH.

The foremost mechanism of PH development includes the functional (hypoxic) pulmonary vasoconstriction, followed by histopathological changes in pulmonary vasculature: proliferation, fibrosis and remodeling. Those histopathological features of SLE-related PH are similar to those of idiopathic PAH [21], but may differ from those in scleroderma-related PAH, which is the most common cause of PAH among autoimmune rheumatic diseases. It is predominantly characterized by endothelial and smooth muscle cell proliferation, as well as fibrinoid necrosis due to vasculitis and deposition of immunoglobulins and complement components in the intimal and medial layers of the pulmonary vessels [21]. (Figure 1). The pathophysiological mechanisms that trigger these functional and structural changes and promote pulmonary vasculature dysfunction and eventually PH occurrence in SLE patients are divided into two main categories:

(A) Immune-mediated and/or inflammatory mechanisms contribute to PAH in SLE patients (Figure 2). This category is less well studied and is associated with a dysregulation of the immune system, the presence of antibodies, and the deposition of inflammatory components in the pulmonary vasculature. Inflammatory cells infiltrate, including macrophages and lymphocytes, have been detected in plexiform lesions (PLs), of the pulmonary vasculature in SLE patients [22,23,24]. A subset of patients with SLE-related PH present with a pulmonary vasculopathy similar to scleroderma-related PAH. It is characterized by a non-inflammatory vascular remodeling and it may contain PLs, associated with anti-U1 ribonucleoprotein (anti-U1 RNP) positivity [25]. PLs, a histologic hallmark of PH, are complex vascular formations derived from remodeled pulmonary arteries [26]. They consist mainly of dysfunctional endothelial cells originating from a misled neo-angiogenesis with an imbalance between apoptosis and endothelium proliferation [26,27]. Autopsies from pulmonary vessels in SLE patients with PAH have shown deposits of antinuclear antibodies (ANA), anti-dsDNA, rheumatoid factor (RF), immunoglobulins and complement, similar to those observed in lupus nephritis [23,28,29]. The presence of these immune complexes within the pulmonary blood vessel walls may be implicated in the pathogenesis of SLE-related PAH [29]. Both immunoglobulins and complement have been demonstrated to deposit on the arterial wall, causing pulmonary vasculitis [19]. Conceivably, in SLE-related PAH, there is a predilection of the immune complexes to bind to larger vessels, whereas in SLE-induced pneumonitis, the smaller vessels may be the main target for immune complex deposition [29]. Moreover, immune complex deposition has been considered a possible pathogenic mechanism in pulmonary veno-occlusion, which is characterized by lesions obstructing pulmonary veins and often presents with a clinical picture similar to PAH [29]. In addition to immune complexes deposition, various growth factors, including growth factors A and B, and the chemokines RANTES/CCL5 and fractalkine/CX3 CL1 are overexpressed in the pulmonary arteries in SLE patients with concomitant severe PAH [23]. The key role of immune system dysregulation in PAH development had been implicated from supporting the potential efficacy of immunosuppressants in SLE-related PAH. However, the current results do not provide robust evidence about the efficacy of immunosuppressive treatment [23]. So, more and larger randomized clinical trials are needed to prove their effectiveness.

A study by Yao et al. [20] explored the genome in SLE-PAH patients and the authors reported that the type I interferon (IFN) response, apoptosis, and protein ubiquitination play a role in the pathogenesis of PAH in these patients [20,21]. Furthermore, they described a repeated gene expression of the T-signaling pathway, demonstrating that the abnormal activation of T-cells plays an important role in inflammation and vascular remodeling in PAH [20]. The ubiquitin–proteasome system (UPS) is a major protein control system that regulates many cellular biological processes, including protein degradation, gene expression and apoptosis [20]. Recent data indicate a possible correlation of UPS abnormalities with pulmonary artery smooth muscle cell proliferation in PAH, where the proteasome inhibitors appeared to be effective to control PAH [20]. The co-expression of genes involved in those pathogenic processes suggests an interplay between them, precipitating their molecular pathways [20]. Another study using type I IFN alpha receptor (IFNAR) knockout mice has explored the role of type I INF in the development of PAH. The type I IFNAR consists of two subunits with different functions: IFNAR1 and INFAR2. They investigated the response to chronic hypoxia of mice without functional IFNAR1. They showed that these mice were protected from the effects of hypoxia on vascular remodeling, right ventricular dysfunction, and elevated serum endothelin-1 (ET-1) [30]. Moreover, other data reported that IFNAR1 plays a role in the release of ET-1. All the aforementioned findings support a pathophysiological relation of type I IFN signaling in PAH development in SLE patients, which may be mediated by ET-1, an IFN-stimulated gene [30]. Additional molecular pathways contributing to SLE-related PAH, may involve an imbalance between vasoconstrictive and vasodilating soluble mediators, leading to pulmonary vasoconstriction and eventually to PVR rise [19,22,31]. ET-1, a pro-proliferative peptide, and thromboxane A2 (TXA-2), a lipid produced by activated platelets, are the main vasoconstrictors and their serum levels are elevated in patients with SLE-related PAH, along severity grade [19]. In about 42% of patients with SLE-related PAH there have been detected antibodies against the endothelin receptor type A [19,31]. Those antibodies promote endothelial dysfunction and inhibit prostacyclin production, one of the main vasodilators, which result in elevated pulmonary artery pressure [19]. Such an imbalance between vasodilators and vasoconstrictors has been reported in idiopathic and scleroderma-related PAH studies [10], while they appear with high ET-1 blood levels. Similar results have been found in SLE patients [32]. ET-1 level correlates with the titer of IgM anti-vascular endothelial cell antibodies (AECA) and the presence of immune complexes in SLE. Therefore, circulating IgM-AECA may stimulate the release of ET-1 from endothelial cells, which in turn will initiate the endothelial damage [32], leading to an immune-mediated vasculopathy [25]. This implicates the contribution of B-cell activation to PAH pathogenesis [25].

(B) Established underlying cardiac or lung disease. PH in SLE patients can result from an underlying diffuse lung disease, such as pulmonary fibrosis, which is not uncommon in SLE, causing hypoxic vasoconstriction [10,33]. The percentage of SLE patients who develop pulmonary complications at some stage in the course of the disease reaches 50–70% [5]. Most SLE patients show signs of lung involvement including disorders in lung parenchyma, pulmonary vessels, pleura and/or diaphragm [9]. Τhe effects on the lung parenchyma usually involve ILD, most commonly chronic interstitial pneumonia and bronchiolitis obliterans with organizing pneumonia (BOOP) progressively leading to fibrosis [9,34]. In pulmonary fibrosis, extensive destruction of the lung parenchyma may occur, including pulmonary vasculature [29]. Pulmonary vein obliteration restricts the capability of the whole pulmonary veins tree to maintain pulmonary blood flow or even to adapt to pressure elevation. Additionally, a pathologic process that may lead to pulmonary veins dysfunction and eventually PH in this population is the presence of recurrent thromboembolic episodes [29]. The chronic persistence of a fibrotic thrombus does not only cause obstructive phenomena in the pulmonary vessels, but it is usually accompanied by endothelial dysfunction, unbalanced fibrinolysis, dysfunctional angiogenesis and immunological mechanisms which can further increase pulmonary artery pressure [35]. SLE patients exhibit a higher risk to thromboembolic events [29], especially those with coexisting antiphospholipid syndrome and seropositivity for lupus anticoagulant, anti-cardiolipin, and beta2 glycoprotein antibodies [25,32]. Other possible etiologies include the presence of left ventricular dysfunction, which usually results from atherosclerotic complications in SLE patients [8]. However, a cardiomyopathy-like left ventricular dysfunction cannot be excluded. Moreover, valvular diseases and the chronic obstructive pulmonary disease (COPD), which are not an exception in SLE, may contribute to high pulmonary pressures and the related pulmonary vessel changes [21].

A good understanding of the underlying pathogenetic mechanisms is required for prompt diagnosis and appropriate targeted therapy of SLE-related PH to prevent irreversible pulmonary vascular damage.

## 4. Diagnosis

The diagnosis of PH in SLE patients should take into account two essential aspects. The first one involves the underestimation of PH, since most physicians are unaware of it. Moreover, PH-linked symptoms, such as dyspnea, non-productive cough, exercise intolerance or fatigue, are frequently present in SLE patients and they are attributed to SLE and its comorbidities. Those common, general symptoms may mask PH development, and so they may delay its investigation and appropriate therapy. The second aspect concerns the identification of underlying cardiac, pulmonary, or vascular disease which is predisposed to PH development. SLE patients may rapidly establish left heart failure, pulmonary disorders, ILD, and chronic thromboembolic disease [36]. Therefore, it is important to have a high index of suspicion for PH among SLE patients. That will lead clinicians to be concerned about and pursue the diagnosis of PH and facilitate its prompt and appropriate management.

### 4.1. Diagnostic Algorithm

Currently, the European Society of Cardiology (ESC) recommends: resting transthoracic echocardiogram (TTE) combined with other tests, including biomarkers, electrocardiography, pulmonary function tests and exercise testing (e.g., cardiopulmonary exercise testing) as annual screening tests for PAH in scleroderma patients [36]. Expanding this diagnostic workup to patients with other CTDs including SLE, TTE remains the first-line, and reliable diagnostic tool for PH and of note is recommended to be performed in the presence of PH-suggesting symptoms or when there are risk factors for PH [36]. TTE provides a non-invasive, measurement of the pulmonary artery systolic pressure (PASP), calculated from the velocity of tricuspid valve insufficiency. It may additionally describe the right and left heart morphology and evaluate cardiac function and valvulopathy [19,36,37]. The assay of certain biomarkers, mainly BNP and NT-proBNP, can help to identify PH in high-risk SLE patients [38]. However, increased levels of natriuretic peptides may also suggest right ventricle dysfunction, a late event in the course of the disease [38]. Above all diagnostic modalities, the RHC is still the gold standard for PH diagnosis and classification [36]. During the procedure, PASP, diastolic pulmonary artery pressure (PAP), pulmonary capillary wedge pressure (PCWP), pulmonary vascular resistance (PVR) and cardiac output (CO) can be accurately measured [19]. Moreover, vasoreactivity can be assessed in order to identify the potential benefit of calcium channel blockers (CCBs) in PH management [36]. Additional imaging and functional tests may be used complementary to identify or exclude other PH etiologies, such as high-resolution tomography to detect ILD, and ventilation/perfusion scan to exclude thromboembolic disease [19]. 

### 4.2. Diagnostic Biomarkers

The term “biomarkers” defines biological molecules, usually found in blood, and indicate an abnormal process or disease at any stage of progression. In the case of natriuretic peptides, both brain natriuretic peptide (BNP) and N-terminal pro-BNP (NT-proBNP) constitute important biomarkers for PH detection in a high-risk population [38]. However, the elevation of their levels may reflect the late stage of PH progression with right ventricle dysfunction [38]. The possible use of other biomarkers for PH diagnosis in SLE, including antiphospholipid antibody (aPL), lupus anticoagulant (LA), anti-cardiolipin (aCL), anti-U1 RNP, endothelin-1 (ET-1), and uric acid (UA) levels, has also been considered in the literature [5,25,38,39]. Elevated UA levels seem to have been most significantly related with PH in SLE patients, and perhaps could be included along with other parameters in a screening protocol for the early detection of the disease [38,39]. Previous studies have supported a possible association of antiphospholipid antibody (aPL), particularly the lupus anticoagulant (LA) [25] and anti-cardiolipin (aCL) [5], as well the anti-U1 RNP, and elevated concentrations of endothelin-1 (ET-1) [25] with the occurrence and/or pathogenesis of PH in SLE [40]. Guo, et al. (2015) [41] suggested ET-1 receptor type A (ETRA) antibodies as a predictive and prognostic serum biomarker of PH in SLE patients. In particular, serum circulating ETRA antibodies were found in 41.5% of patients with SLE-related PH compared to 17% of SLE patients without PH [41]. Similarly, many studies support that SLE patients with PH are more likely to be positive for aPL than SLE patients without PH [40,42]. However, aPLs have been detected in PH-patients without SLE [43], and there is a lack of strong evidence to support their possible role in PH pathogenesis among SLE patients. A meta-analysis of 31 studies (Zuily S, et al. 2017) [44] demonstrated a two-fold higher risk for PH in SLE patients with aPLs than in those without PH. Besides this, the risk of PH development varied along with aPL types. Although LA and IgG aCL have been linked to a significantly higher risk of PH development in SLE, the presence of IgM aCL and anti-beta 2-glycoprotein I (aβ2-GPI) was not significantly associated with PH [44]. Furthermore, the CSTAR cohort study (Qu et al. 2021) [45] that included 3624 SLE patients reported that anti-RNP antibodies and anti-SSA/SSB antibodies are strong predictors for PH in SLE. Another study of 74 SLE patients has shown a higher percentage of seropositivity of rheumatoid factor (RF) in SLE patients with PH than in those without PH [46]. Other factors, including the presence of anti-ET-1 [47], anti-U1 RNP [14], anti-topoisomerase I (anti-Scl-70) [48], anti-SmD1 [49], and anti-Smith (Sm) [50], low erythrocyte sedimentation rate (ESR) [18] and hypocomplementemia [50] have also been associated with the presence of PH in SLE. Thus, it would be clinically meaningful to apply serum biomarkers for the early detection of PH in SLE patients. However, their role in PH remains unclear and more studies are needed to validate their usage. 

In addition to the aforementioned immune-mediated mechanisms, a cross sectional study of 114 SLE patients (Kim et al. 2014) [38] showed significantly higher serum uric acid (UA) levels in SLE patients with PH than in those without PH. Moreover, UA levels had a positive correlation with pulmonary artery pressure. It was also shown that UA levels in patients with SLE-PH were higher compared to patients with PH in the context of underlying lung disease. Therefore, it is recommended to perform NT-proBNP measurement and echocardiography in SLE patients with unexplained high UA levels. Moreover, the persistent elevation of plasma UA (≥7 mg/dL) in patients with normal pulmonary artery pressures at baseline may predict the development of PH during a 7 year follow-up (Castillo-Martínez, et al. 2016) [39]. Hence, high plasma UA levels may be able to distinguish SLE patients at risk for PH development [51]. Table 1 summarizes the role of biomarkers in SLE patients with PH (Table 1).

SLE patients may have few and non-specific signs and symptoms associated with PH. Its onset is often insidious, but by the time of diagnosis, irreversible damage in pulmonary vasculature has already been established. Therefore, a “gold-standard” biomarker or screening protocol for early detection of PH in SLE patients is deemed necessary. 

## 5. Management

According to the recently published guidelines by the ESC and the European Respiratory Society, the therapeutic approach to patients with SLE-related PH does not differ from that of idiopathic PAH and includes escalating intensive pharmaceutical treatment [36]. Referral to a specialized medical center is highly recommended when available. General measures include oxygen supply, light exercise training and diuretics, depending on the clinical status of each patient [52].

A meta-analysis evaluating the need for anticoagulation in CTD-associated PAH showed no reduction in mortality among patients receiving oral anticoagulants [40]. However, the meta-analysis mainly included patients with systemic sclerosis, and the extrapolation of its findings to SLE patients should be made with caution [53]. A retrospective study of 663 patients with non-idiopathic PAH, including 168 patients with CTD, assessed the response to acute vasodilator testing and long-term response to calcium channel blocker (CCB) therapy. Altogether, 10.1% (*n* = 17) of the patients with CTD had a positive response to acute vasodilator testing, and only one patient (0.6%) was considered a long-term responder to CCB, with hemodynamic and clinical improvement remaining in NYHA class I- II after 1 year’s treatment [54].

Immunosuppressive therapy seems to be necessary in all patients with SLE-related PH. In a small retrospective study of 24 patients with SLE, most patients with a favorable baseline NYHA functional class had a positive response to immunosuppressive therapy [55]. In another randomized control trial of 34 individuals with SLE-related PH, the investigators compared the efficacy of cyclophosphamide (at doses 0.5–0.6 g/m^2^) and enalapril (10 mg/d). Cyclophosphamide had a significant effect on PASP reduction after 6 months of treatment and led to improvement of the NYHA functional class, while enalapril had no effect [28]. Another small retrospective study assessed the current treatment of PAH in 23 patients with CTD, among them 13 patients with SLE [14]. All patients were originally treated with intravenous cyclophosphamide and oral prednisone, while seven of them received additional pulmonary vasodilators, including bosentan, epoprostenol and subcutaneous treporostinil. Although this is a very small study, it strongly indicated the clinical benefits of immunosuppressants in most SLE patients and pulmonary vasodilators as an add-on therapy, especially among non-responders to immunosuppressants [23].

It has been hypothesized that pulmonary vasodilators may be required, especially in more severe PAH disease, based on invasive measurements or functional class assessment (NYHA III or IV). A randomized controlled trial evaluated the effects of ambrisentan monotherapy, tadalafil monotherapy and combination therapy in patients with CTD-associated PAH (9% with SLE). Ambrisentan, an endothelin-1 receptor antagonist, was administered at 5 mg/day and then increased to 10 mg/day. Tadalafil, a phosphodiesterase 5 inhibitor, was administered at 20 mg/day and then increased to 40 mg/day. The combined therapy conferred better outcomes than each monotherapy and its effects associated with baseline hemodynamic measurements, such as high pulmonary vascular resistance, low cardiac index and low pulmonary arterial wedge pressure, and with the absence of signs of both left heart disease and restrictive lung disease. Although combined therapy slowed down the PAH progression, it failed to prevent PAH development at all. The small number of patients cannot provide safe conclusions for the SLE group [56]. Sildenafil, a known phosphodiesterase 5 inhibitor, was evaluated in a randomized control trial among patients with CTD-associated PAH. Sildenafil was administered at doses of 20 mg, 40 mg and 80 mg 3 times/day to 19 SLE patients. The low dose (20 mg t.i.d.) adequately improved functional and hemodynamic parameters in SLE patients with PAH [57].

Riociguat is a soluble guanylate cyclase stimulator and its efficacy was evaluated in a randomized controlled trial with a total of 443 patients with CTD-related PAH. A small proportion of them (*n* = 18) had SLE. At the end of 2 years of follow-up, the clinical and hemodynamic status of the riociguat group improved or remained stable [58], but the effect in the SLE subgroup is unknown. Another promising treatment for SLE-related PAH are prostacyclin analogs, such as treprostinil and epoprostenol. The efficacy of subcutaneous administration of treprostinil was evaluated using a randomized control trial including 25 patients with SLE. It demonstrated improvement in the following parameters: walking capacity, dyspnea fatigue and hemodynamic measurements, in a dose dependent-manner [59]. A case series of six individuals with SLE-related PAH reported improvement in symptoms and hemodynamic measurements after chronic treatment with intravenous epoprostenol ranging from 4 to 46 ng/kg/minute [60]. Selexipag is a selective prostacyclin receptor agonist that was administered to 84 SLE patients with PAH, at doses of 200–1600 μg twice daily and seemed to delay the progression of the disease according to this randomized control trial [61].

In the case of PH secondary to underlying concomitant diseases (e.g., heart failure or ILD), the proposed therapy in SLE patients does not differ from that already applied in patients with PH, but without SLE.

It is worth noting that few small-scale, randomized clinical trials have been published examining the efficacy of the available treatments for SLE-related PH. The current evidence has low levels of evidence to develop a specific therapeutic algorithm in SLE patients. Unambiguously, more studies need to be conducted by recruiting a larger number of SLE patients, aiming to identify whether immunosuppressive therapy targeting prevents PH development or improves the symptoms of established PH.

## 6. Prognosis

A retrospective study in 51 patients with SLE-related PAH reported 3-year and 5-year survival rates of 89.4% and 83.9%, respectively, while the presence of anti-U1-RNP antibodies was associated with an improved prognosis [62]. According to this study, the median time between SLE diagnosis and PH diagnosis was 4.9 years [62]. Older studies have documented lower ranges of 5-year survival rates (60.2% and 85%) and 3-year survival rates (range from 74 to 88%) [40,63,64,65]. Among various prognostic factors, renal involvement, higher pulmonary vascular resistance and alkaline phosphatase (ALP) are independently associated with death [62,65], while Raynaud’s phenomenon constitutes a predictor of increased survival [40]. Overall, it is evident that PAH presence negatively affects the survival of individuals with SLE.

## 7. Conclusions

SLE is a chronic autoimmune disease affecting virtually any system. Pulmonary involvement is a common manifestation, including the PH. Survival rates for patients with SLE-related PAH vary between studies and 5-year survival rates are estimated between 60.2% and 85%. Although the exact etiology of PAH due to SLE per se is still unknown, some pathogenic mechanisms have been proposed. In addition to this, PH development in SLE patients may be secondary to cardiorespiratory and/or thromboembolic diseases. Presumably, the etiopathogenic mechanisms include cardiorespiratory disorders, thromboembolic vessel damage and immune system dysregulation with the deposition of inflammatory cells and autoantibodies in the pulmonary vasculature. SLE-related PH is often diagnosed late, when irreversible damage has occurred. Prompt identification of SLE-related PH is vital and may facilitate the early initiation of appropriate targeted therapy in the course of the disease. There is no established diagnostic tool or screening protocol to predict the development of PH in SLE. The management of PH in SLE patients is similar to idiopathic PAH. Anticoagulation is not deemed to be necessary for PH unless other indications exist. Treatment options include immunosuppressive agents, phosphodiesterase type 5 inhibitors, endothelin receptor antagonists, prostacyclin pathway agonists and soluble guanylate cyclase stimulants. Notably, randomized placebo control trials need to be conducted in order to investigate the efficacy of each drug class.

## 8. Patents

This section is not mandatory but may be added if there are patents resulting from the work reported in this manuscript.

## Figures and Tables

**Table 1:** Published studies investigating the role of biomarkers in SLE patients with pulmonary hypertension.

Reference	Studies—Cohorts	Study Design	Outcomes
Lian, et al., 2012 [14]	147 SLE-pts 41 pts w/ PAH 106 pts w/o PAH	Retrospective case-control study, Duration: 9 years	PAH correlation: ↑ high disease activity ↑ aCL, anti-U1 RNP Presence of serositis, Reynaud’s phenomenon
Guo, et al., 2015 [41]	Exploratory cohort: 156 SLE-pts: 76 w/PAH 80 w/o PAH Validation cohort: 164 SLE-pts: 82 w/PAH 82 w/o PAH	Case-control study	↑ ETRA antibodies in SLE-pts w/PAH
Cefle, et al., 2011 [42]	107 SLE-pts: 10 w/ PH (PASP > 30 mmHg) 97 w/o PH	Case-control study	SLE-pts w/ PH: ↑ aPL, aCL ANA, anti-dsDNA ↓ serum complement level
Min, et al., 2015 [40]	154 Korean pts 35 SLE-pts w/ PH 119 SLE-pts w/o PH	Retrospective cohort study. Relation of clinical and laboratory characteristics w/ mortality	Mortality correlation: Presence of PH ↑ lupus nephritis ↓ serum complement level ↑ aCL in SLE-PH (no significant correlation w/ mortality)
Zuily, et al., 2017 [44]	31 studies, 4480 SLE-pts	Systematic review and meta-analysis, evaluation of the risk for PH development	↑ risk for PH: ↑ LA, aCL IgG aβ2-GPI, aCL IgM
Qu, et al., 2021 [45]	3624 SLE-pts 92 pts developed PAH	Prospective cohort study, risk stratification model for PAH development Median follow up: 4.84 years	↑ risk for PAH: mild ILD ↓ renal involvement serositis
Chow, et al., 2012 [47]	23 studies including SLE-pts w/ PH	Systematic review, for evaluation of possible prognostic factors	↓ survival correlation: ↑ PASP Presence of Reynaud’s phenomenon ↑ aCL ↑ presence of plexiform lesions
Gussin, et al., 2001 [48]	128 SLE-pts	Retrospective cohort study	↑ risk for PAH: ↑ anti–Scl-70
Hu, et al., 2017 [49]	269 Chinese SLE-pts: 100 drug naïve pts 169 drug-treated	Cross sectional study	↑ anti-SmD1 correlation w/: PAH malar rash nonscarring alopecia hypocomplementemia seizures renal disorders
Mizus, et al., 2019 [50]	154 SLE-pts w/ PAH	Cross sectional study	↑ prevalence of PAH in: African- American ↑ aPL, anti-Ro, anti-La ↑ serological activity
Huang, et al., 2016 [18]	111 SLE-pts w/PAH	Cross sectional study	↑ risk for PAH: Presence of pericardial effusion ↑ anti-RNP, anti-SSA, UA Presence of ILD
Kim, et al., 2015 [38]	114 Korean SLE-pts: 84 pts w/ sPAP < 30 mmHg 21 pts w/ sPAP =30–39 mmHg	Cross-sectional study	↑ risk for PAH: ↑ SLE disease activity index score UA≥ 6.5 mg/dl ↓ Anti-Ro/SS-A LA, aCL, aβ2-GPI
Castillo-Martínez, et al., 2016 [39]	44 SLE pts: 10 pts w/ PAH 34 pts w/o PAH	Prospective cohort study Follow-up: 7-years	↑ risk for PAH: UA≥ 7 mg/dl

aβ2-GPI: anti-beta 2 glycoprotein I antibodies; Abs: antibodies; aCL: anticardiolipin antibodies; ANA: antinuclear antibodies; anti-dsDNA: anti-double stranded DNA antibodies; anti-U1 RNP: Anti-U1-Ribonucleoprotein antibodies; anti–Scl-70: anti-scleroderma 70 antibodies; anti-SmD1: anti-Smith D1; aPL: antiphospholipid antibodies; a-SMA: alpha smooth muscle actin; ELISA: enzyme-linked immunosorbent assay; ET-1: endothelin-1; ETRA: ET-1 receptor type A antibodies; HUVECs: human umbilical vein endothelial cells; ILD: interstitial lung disease; LA: lupus anticoagulant; PAH: pulmonary artery hypertension; PDGFB: platelet derived growth factor subunit B; PDGFRb: platelet-derived growth factor receptor beta; PH: pulmonary hypertension; PASMCs: Human pulmonary artery smooth muscle; pts: patients; RHC: right heart catheterization; SLE: systemic lupus erythematosus; sPAP: systolic pulmonary artery pressure; UA: uric acid; VEGF-A: vascular endothelial growth factor A; w/ = with; w/o: without; 5-HTT: serotonin transporter.
